# Gut-Spleen Axis: Microbiota via Vascular and Immune Pathways Improve Busulfan-Induced Spleen Disruption

**DOI:** 10.1128/msphere.00581-22

**Published:** 2022-12-13

**Authors:** Hanhan Fang, Xiaohui Feng, Tao Xu, Ruqing Zhong, Dongxin Lu, Hongfu Zhang, Wei Shen, Yong Zhao, Liang Chen, Junjie Wang

**Affiliations:** a College of Life Sciences, Qingdao Agricultural University, Qingdao, People’s Republic of China; b State Key Laboratory of Animal Nutrition, Institute of Animal Sciences, Chinese Academy of Agricultural Sciences, Beijing, People’s Republic of China; c College of Science, Health, Engineering and Education, Murdoch University, Perth, Australia; University of Michigan-Ann Arbor

**Keywords:** fecal microbiota transplantation (FMT), spleen, vascularization, immune, iron homeostasis

## Abstract

Fecal microbiota transplantation (FMT) is an effective means of modulating gut microbiota for the treatment of many diseases, including Clostridioides difficile infections. The gut-spleen axis has been established, and this is involved in the development and function of the spleen. However, it is not understood whether gut microbiota can be used to improve spleen function, especially in spleens disrupted by a disease or an anti-cancer treatment. In the current investigation, we established that alginate oligosaccharide (AOS)-improved gut microbiota (A10-FMT) can rescue anticancer drug busulfan-disrupted spleen vasculature and spleen function. A10-FMT improved the gene and/or protein expression of genes involved in vasculature development, increased the cell proliferation rate, enhanced the endothelial progenitor cell capability, and elevated the expression of the cell junction molecules to increase the vascularization of the spleen. This investigation found for the first time that the reestablishment of spleen vascularization restored spleen function by improving spleen immune cells and iron metabolism. These findings may be used as a strategy to minimize the side effects of anti-cancer drugs or to improve spleen vasculature-related diseases.

**IMPORTANCE** Alginate oligosaccharide (AOS)-improved gut microbiota (A10-FMT) can rescue busulfan disrupted spleen vasculature. A10-FMT improved the cell proliferation rate, endothelial progenitor cell capability, and cell junction molecules to increase vasculature formation in the spleen. This reestablishment restored spleen function by improving spleen immune cells and iron metabolism. These findings are useful for the treatment of spleen vasculature-related diseases.

## INTRODUCTION

The spleen is an important lymphoid organ that removes aging erythrocytes, recycles iron, elicits immunity, and supplies erythrocytes after a hemorrhagic shock (1, 2). For many reasons, the global incidence of human malignancies continues to rise (3, 4), and this has resulted in an increased use of anti-cancer drugs (5, 6). Busulfan is an anti-cancer alkylating agent that has been widely used in the treatment of lymphoma and chronic leukemia, and it has also been used to improve outcomes after allogeneic hemopoietic cell transplantation or as a conditioning regimen before hematopoietic stem cell transplantation (5–7). Busulfan is one of the few anti-cancer drugs that is used in children who are 3 years old or younger (6–8). It gives excellent results, but it also produces several side effects (5, 7), one of which is toxicity to the spleen (9). Busulfan can cause spleen lesions and a decrease in the number of hematopoietic cells (9). Despite busulfan being such an important anti-cancer drug, especially for children, it does have side effects on the spleen and other organs. Therefore, it is critical to employ good drug management to minimize any negative impacts.

The gut microbiota is reported to influence many aspects of our health because it provides nutrients and vitamins, prevents pathogen colonization, and maintains homeostasis of the epithelial mucosa and immune system (10). Gut microbes have been implicated in cancer therapy, modulated chemotherapy efficacy, and the toxicity of several chemotherapy agents (2, 3, 10). Moreover, we found that the anticancer drug busulfan disturbs the gut microbiota to diminish spermatogenesis (11, 12). Gut mucosal bacteria play a vital role in the development and maturation of the spleen (13–17). The gut microbiota-spleen axis plays important roles in modulating the systemic inflammation process (15). The restoration of gut microbiota to a health state using fecal microbiota transplantation (FMT) is a novel, key therapeutic approach that is of growing interest; this procedure is continuing to gain importance in clinical and research settings (18). Although FMT is primarily used to cure recurrent Clostridioides difficile infections (19), it has also been trialed for treating food allergies (20), increasing health and life spans (19), improving spermatogenesis and male fertility (11, 12), and preventing intestinal injury (21, 22). The effect of FMT on spleen disruption is an emerging novel area of interest, as the spleen is an important organ in the defense of human health.

Alginate oligosaccharides (AOS), linear polymers composed of β-d-mannuronate (M) and α-l-guluronate (G) (23), have attracted considerable pharmaceutical attention because they possess anti-inflammatory (24), anti-apoptosis (25), antiproliferation (26), and antioxidant activities (27–29) as well as anti-cancer effects (30). AOS inhibits the neuroinflammation induced by lipopolysaccharide/β-amyloid (Aβ) via the reduction of nitric oxide, prostaglandin E2, proinflammatory cytokines, and nuclear factor (NF)-κB production (31). Moreover, AOS can decrease chemotherapeutic agent doxorubicin-caused acute cardiotoxicity by inhibiting oxidative stress and endoplasmic reticulum stress-mediated apoptosis (30, 32). Recently, we found that AOS increase intestinal beneficial microbes to improve blood metabolites and ameliorate spermatogenesis and male fertility in busulfan-treated animals (33). Moreover, the FMT of AOS-improved gut microbiota (AOS dose animal intestinal content) increases the beneficial microbes in the receipt animals and improves the blood and testis metabolites to ameliorate spermatogenesis and to elevate male fertility in busulfan-treated, high-fat diet-fed, type 1 and type 2 diabetic animals (11, 12, 34–36). In a preliminary study, we found that the FMT of AOS-improved microbiota can improve busulfan-induced spleen disruption. This study was designed to explore the beneficial effects of AOS-improved gut microbiota on busulfan-impaired spleen functions. This is a parallel study that is published early (11), wherein the FMT of AOS-benefited microbiota improved spermatogenesis.

## RESULTS

### A10-FMT improved busulfan-disrupted spleen cells.

**FIG 1:**
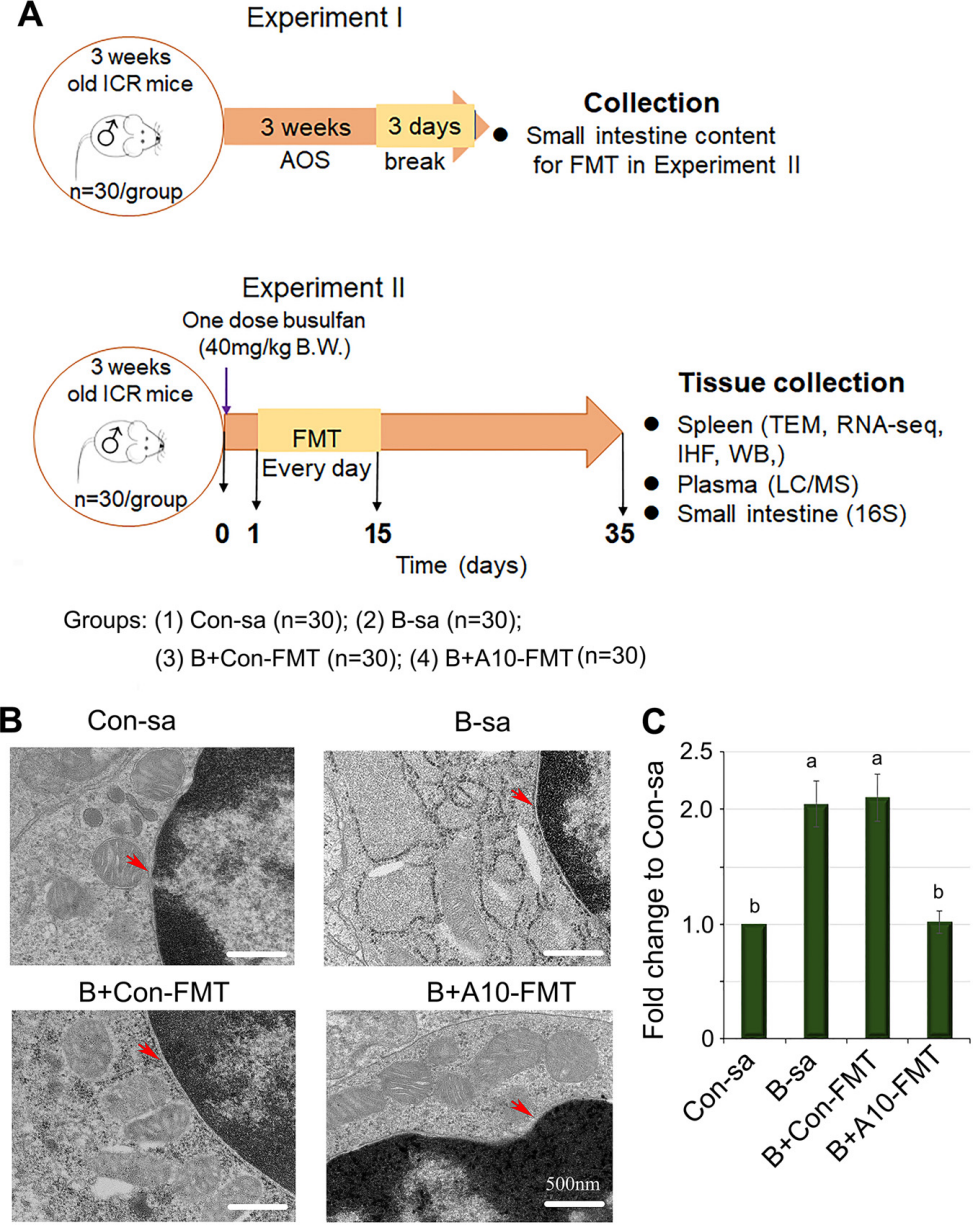
Busulfan disrupted mouse spleen. (A) Study design. (B) TEM analysis showing the A10-FMT rescued spleen cellular disruption caused by busulfan. The red arrows indicate the gap between the nuclei and the cytoplasm. (C) The quantitative data for the gap between the nuclei and the cytoplasm (red arrow indication in panel B). The *y* axis was the fold change to the Con-sa group. The *x* axis represents the treatments. Note: the a and b notation indicate statistically significant differences between groups with *P* < 0.05 and *n* = 4.

To determine the beneficial advantages of FMT on the busulfan-disrupted spleen, mice were first treated with busulfan and then subjected to FMT administration. There were four treatment groups: the control group (Con-sa, mice dosed with saline), B-sa (busulfan treatment plus dosing with saline), B+Con-FMT (busulfan treatment plus dosing with FMT from control mice), and B+A10-FMT (busulfan treatment plus dosing with FMT from alginate oligosaccharides, 10 mg/kg dosed mice) (Fig. 1A). Compared to the control, busulfan administration damaged spleen cells by causing swelling of the endoplasmic reticulum (ER) and mitochondria; furthermore, this led to a gap between the nuclei and the cytoplasm (indicated by a red arrow in Fig. 1B). FMT from the control mice (Con-FMT) partially rescued busulfan-caused ER and mitochondrial disruption; however, it was unable to reduce the gap between the nuclei and the cytoplasm (Fig. 1B). FMT from the AOS-dosed mice (A10-FMT) significantly rescued spleen cells, almost totally restoring the ER and mitochondrial swelling, and removed the gap between the nuclei and the cytoplasm (Fig. 1B).

**FIG 2:**
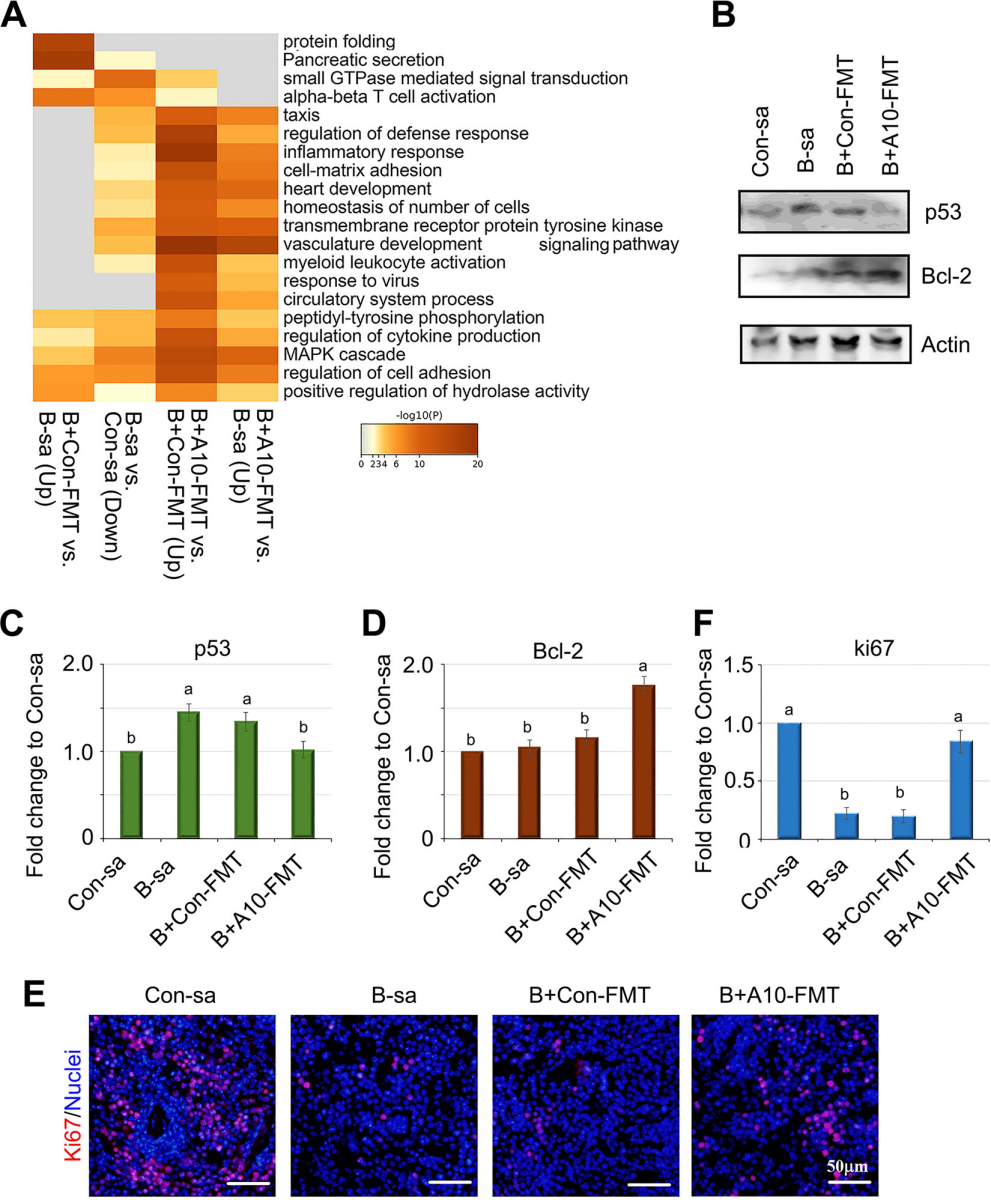
Busulfan disturbed the gene expression and cell survival in mouse spleens. (A) Enrichment analysis of RNA-seq data showing a comparison of differentially expressed gene functions. “Up”, gene expression increased; “Down”, gene expression decreased (*n* = 3 per group). (B) WB analysis showing cell apoptosis-related protein changes (*n* = 3 per group). (C) Quantitative data for p53. (D) Quantitative data for Bcl-2. (E) IHF staining for Ki67 showed the spleen cell proliferation status (*n* = 4 per group). (F) Quantitative data for Ki67. For panels C, D, and F, the *y* axis was the fold change of the Con-sa group. The *x* axis represents the treatments. Note: the a and b notation indicate statistically significant differences between groups with *P* < 0.05.

To identify potential A10-FMT rescued genes, we compared the global gene expression profiles of the spleen tissue after the treatments. The gene expression profiles for these four treatments were different and were easily separated (Fig. S1A–E). A functional enrichment analysis of the differentially expressed genes revealed multiple interesting phenomena (Fig. 2A). Compared to the control group, busulfan decreased many genes that were enriched in the pathways of “Vasculature development”, “Homeostasis of number of cells”, “Cell-matrix adhesion”, “Regulation of defense response”, and “Myeloid leukocyte activation”, whereas these genes were increased by B+A10-FMT versus B-sa but not by B+Con-FMT versus B-sa (Fig. 2A). The data indicated that busulfan disturbed the vascularization and immune response of the spleen, which could be rescued by A10-FMT. At the same time, busulfan induced cell apoptosis by increasing the level of the protein p53 and decreased the cell proliferation rate by reducing the number of ki67 positive cells, and all of these phenomena were restored by A10-FMT (Fig. 2B–D).

10.1128/msphere.00581-22.1 FIG S1 RNA-seq data summary for the mouse spleens. (A) The PCA analysis used in the RNA-seq analysis. (B) The KEGG enrichment analysis for the genes decreased in the comparison of B-sa versus Con-sa. (C) The KEGG enrichment analysis for the genes increased in the comparison of B+A10-FMT versus B-sa. (D) The KEGG enrichment analysis for the genes increased in the comparison of B+Con-FMT versus B-sa. (E) The KEGG enrichment analysis for the genes increased in the comparison of B+A10-FMT versus B+Con-FMT. Download FIG S1, TIF file, 2.9 MB. Copyright © 2022 Fang et al. 2022 Fang et al. https://creativecommons.org/licenses/by/4.0/ This content is distributed under the terms of the Creative Commons Attribution 4.0 International license.

### A10-FMT restored busulfan-disturbed spleen vascularization.

**FIG 3:**
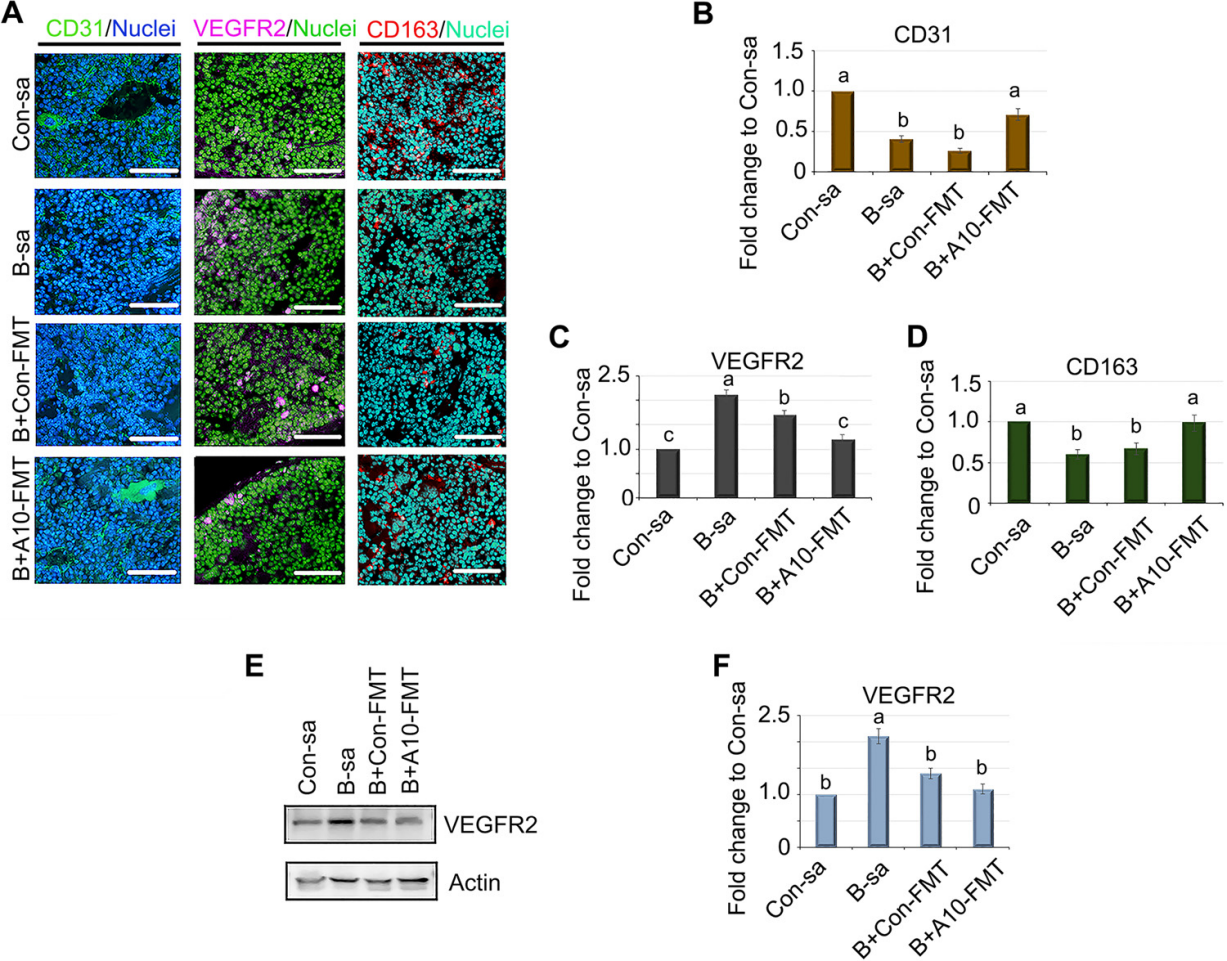
A10-FMT rescued busulfan disrupted spleen vasculature formation. (A) IHF staining for the protein levels of CD31, VEGFR2, and CD163 (*n* = 4 per group). (B) Quantitative data of IHF for CD31. (C) Quantitative data of IHF for VEGFR2. (D) Quantitative data of IHF for CD163. (E) WB analysis for VEGFR2 (*n* = 3 per group). (F) Quantitative data of WB for VEGFR2. For panels B–D and F, the *y* axis was the fold change of the Con-sa group. The *x* axis represents the treatments. Note: the a, b, and c notation indicate statistically significant differences between groups with *P* < 0.05.

The CD31 (platelet and endothelial cell adhesion molecule 1, PECAM-1) blood vessel marker is expressed on vascular endothelial cells in both the white pulp (WP) and the red pulp (RP) of the spleen (37, 38). Busulfan decreased the number of CD31 positive cells in spleen tissues, and this number was restored by B+A10-FMT but not by B+Con-FMT (Fig. 3A and B). Endothelial progenitor cells (EPC) are a type of bone marrow (BM)-derived progenitor cell; they circulate throughout the body in the blood and have the capacity to differentiate into endothelial cells (38, 39). Vascular endothelial growth factor receptor-2 (VEGFR2; Flk-1) is an endothelial marker protein of EPC (37, 38). Busulfan increased the number of VEGFR2 positive cells, whereas A10-FMT restored the cells to control levels (Fig. 3A and B). The protein level of VEGFR2 was verified via Western blotting (WB) analysis (Fig. 3C and D). The data suggest that busulfan disrupted the spleen vasculature, which recruited more EPC to differentiate into endothelial cells. The data were confirmed by the expression of the hemoglobin scavenger receptor (CD163) protein in the spleen (Fig. 3A and B). CD163 plays major roles in the clearance and endocytosis of hemoglobin/haptoglobin complexes (40). Busulfan decreased the CD163 protein levels in the spleen, and these were recovered by B+A10-FMT (Fig. 3A and B). At the same time, the gene expression of CD163 followed the same trend as did the protein expression (Fig. 3E).

**FIG 4:**
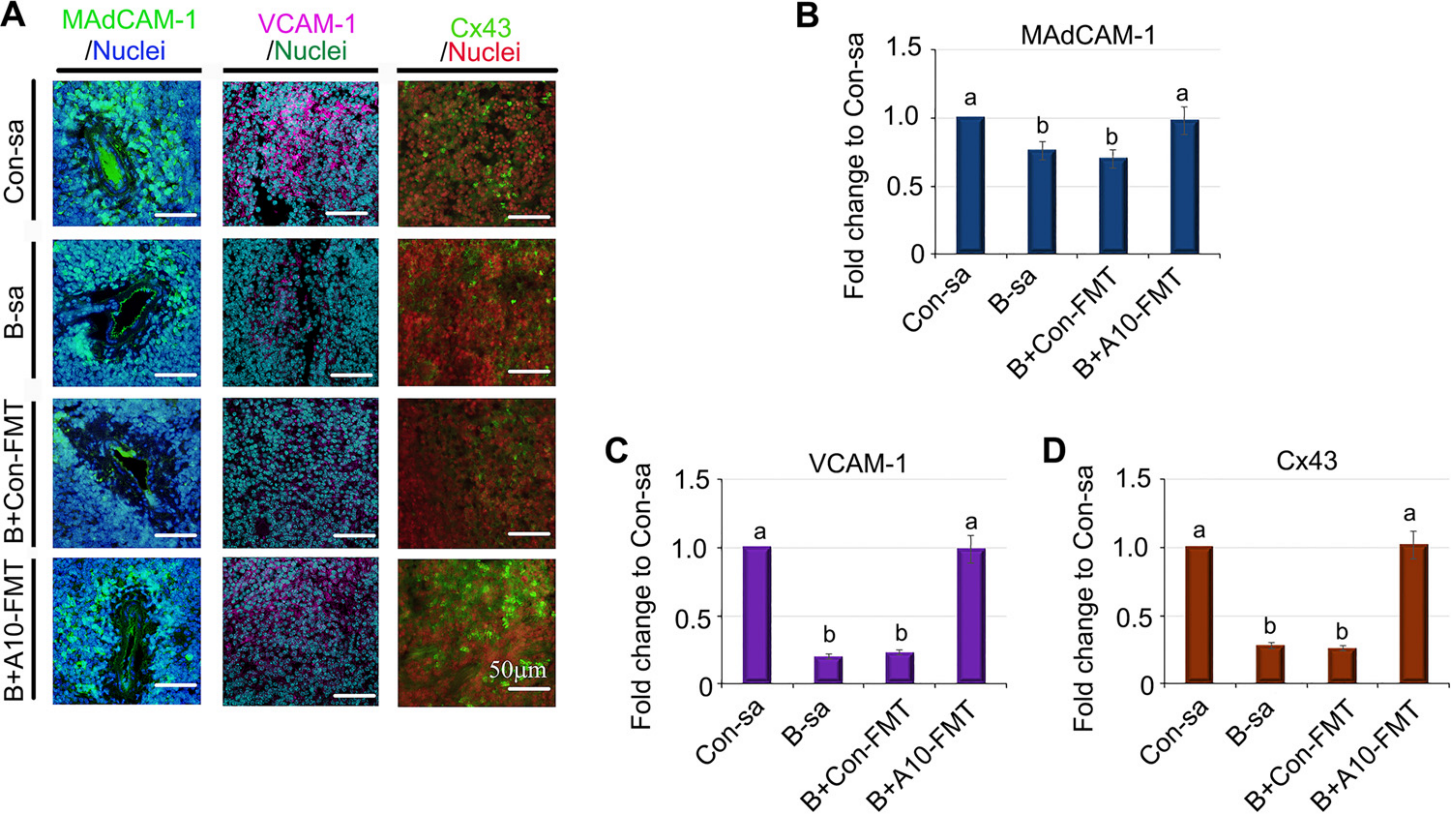
A10-FMT restored the protein levels of MAdCAM-1, VCAM-1, and Cx43, which were upset by busulfan. (A) IHF staining for the protein levels of MAdCAM-1, VCAM-1, and Cx43 (*n* = 4 per group). (B) Quantitative data of IHF for MAdCAM-1. (C) Quantitative data of IHF for VCAM-1. (D) Quantitative data of IHF for Cx43. For panels B–D, the *y* axis was the fold change of the Con-sa group. The *x* axis represents the treatments. Note: the a and b notation indicate statistically significant differences between groups with *P* < 0.05.

Mucosal vascular addressin cell adhesion molecule 1 (MAdCAM-1) is suggested to contribute to the marginal sinus (MS) organization of developing spleen endothelial cells (37). The expression of MAdCAM-1 was reduced by busulfan; however, this was restored by B+A10-FMT (Fig. 4A and B). The data further confirmed that spleen vasculature was disrupted by busulfan and that A10-FMT was capable of restoring it.

During mammalian embryogenesis, integrin alpha-4/beta-1 (VLA-4) is predominantly expressed in EPC (41, 42). Moreover, in mammalian cells, vascular cell adhesion molecule-1 (VCAM-1) is the major ligand for VLA-4 (43). It has been demonstrated that the VCAM-1^+^ macrophage guides the homing of EPC to a vascular niche (42). In the current investigation, busulfan decreased the number of VCAM-1 positive cells, which was increased by A10-FMT (Fig. 4A and B).

Connexins (Cxs) are structural proteins that form gap junctions (Gjs). These junctions control important immunity-regulating cellular processes. VEGF promotes the differentiation of EPCs and vascular repair through Cx43 (44, 45). It has been shown that Cx43 plays important roles in the activation of spleen cells and in immunoglobulin production (44). Furthermore, Cx43 is also involved in EPC differentiation and in vascular repair in spleen cells (45). Busulfan decreased the protein level of Cx43 (as well as the number of Cx43 positive cells), which was restored by A10-FMT but not by Con-FMT (Fig. 4A and B). Taken together, these data suggest that A10-FMT is able to promote vascularization in the spleen.

### A10-FMT recovered busulfan-upset spleen functions.

**FIG 5:**
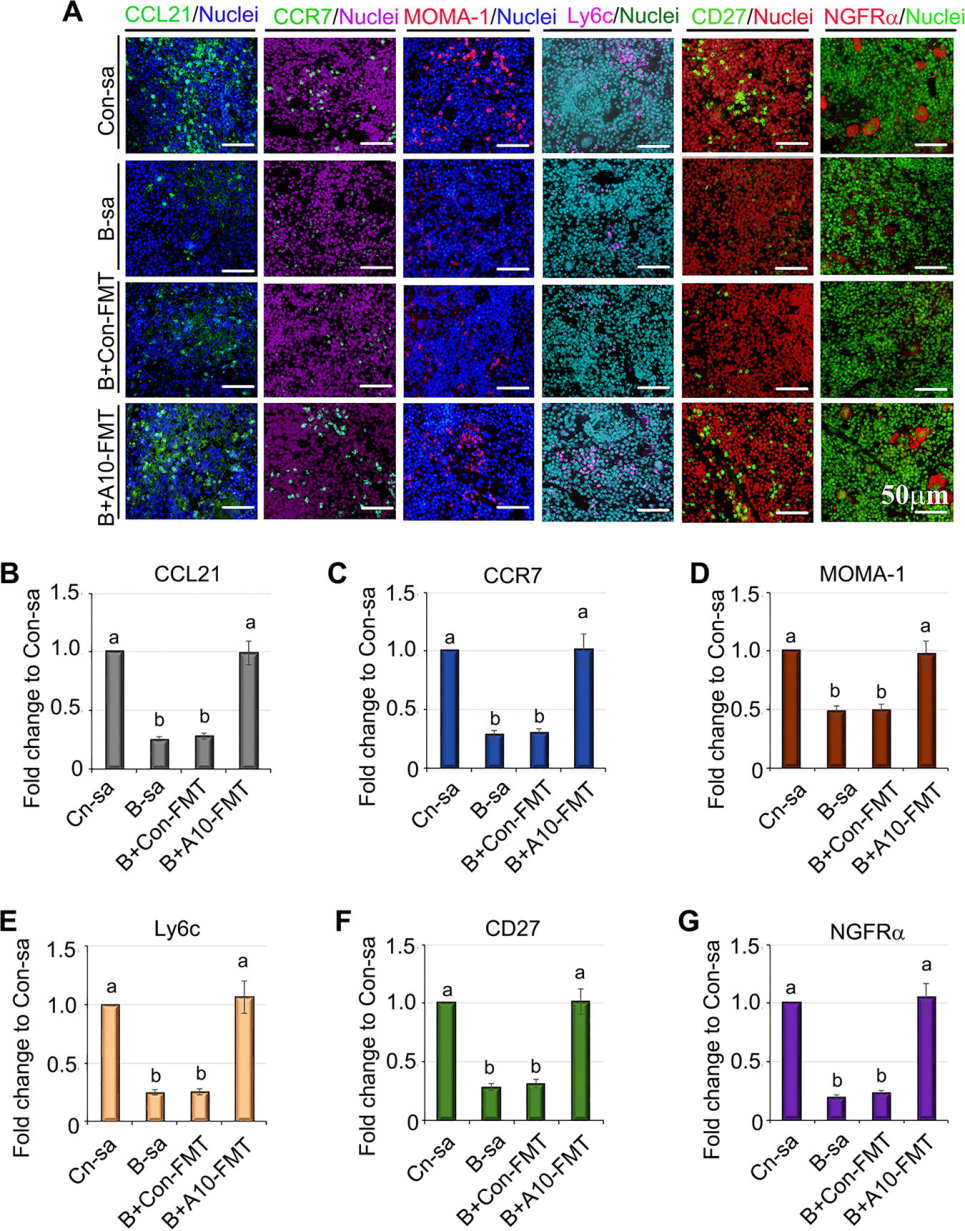
A10-FMT improved the different kinds of immune cells via the rescue of the protein levels of the marker genes. (A) IHF staining for the protein levels of CCL-21, CCR7, MOMA-1, Ly6C, CD27, and NGFRα (*n* = 4 per group). (B) Quantitative data of IHF for CCL-21. (C) Quantitative data of IHF for CCR7. (D) Quantitative data of IHF for MOMA-1. (E) Quantitative data of IHF for Ly6C. (F) Quantitative data of IHF for CD27. (G) Quantitative data of IHF for NGFRα. In panels B and G, the *y* axis was the fold change of the Con-sa group. The *x* axis represents the treatments. Note: the a, b, and c notation indicate statistically significant differences between groups with *P* < 0.05.

Since A10-FMT restored spleen vasculature formation, we set out to establish its beneficial advantages with respect to spleen immune function. To do this, the statuses of different types of spleen immune cells were determined (Fig. 5). T-cell protein CCL21 and its receptor CCR7 play important roles in maintaining the active migratory states of T cells (1). Busulfan decreased the protein levels of CCL21 and CCR7 in the spleen, and these were increased by A10-FMT but not by Con-FMT (Fig. 5A and B). Marginal zone (MZ) B cells have a circulating counterpart and express the memory molecule CD27 (46, 47). Busulfan decreased the number of CD27 positive cells in the spleen, and this number was restored by A10-FMT but not by Con-FMT (Fig. 5A and B). MOMA-1 is the marker of metallophilic macrophage cells and is located peripheral to the MS in the MZ. Busulfan decreased the number of MOMA-1 positive cells in the spleen, and this number was restored by A10-FMT but not by Con-FMT (Fig. 5A and B). Splenic monocytes resemble their blood counterparts morphologically in the expression of Ly6C (2). Busulfan decreased the number of Ly6C positive cells in the spleen, and this number was restored by A10-FMT but not by Con-FMT (Fig. 5A and B). At the same time, the gene expression of MOMA-1 and Ly6C followed the same trends as did their protein levels (Fig. 5C). NGFR (Nerve growth factor receptor; CD271) is an optimal target for the immunohistological detection of follicular dendritic cells (FDCs) (48). Busulfan decreased the number of NGFR positive cells in the spleen, and this number was restored by A10-FMT but not by Con-FMT (Fig. 5A and B).

**FIG 6:**
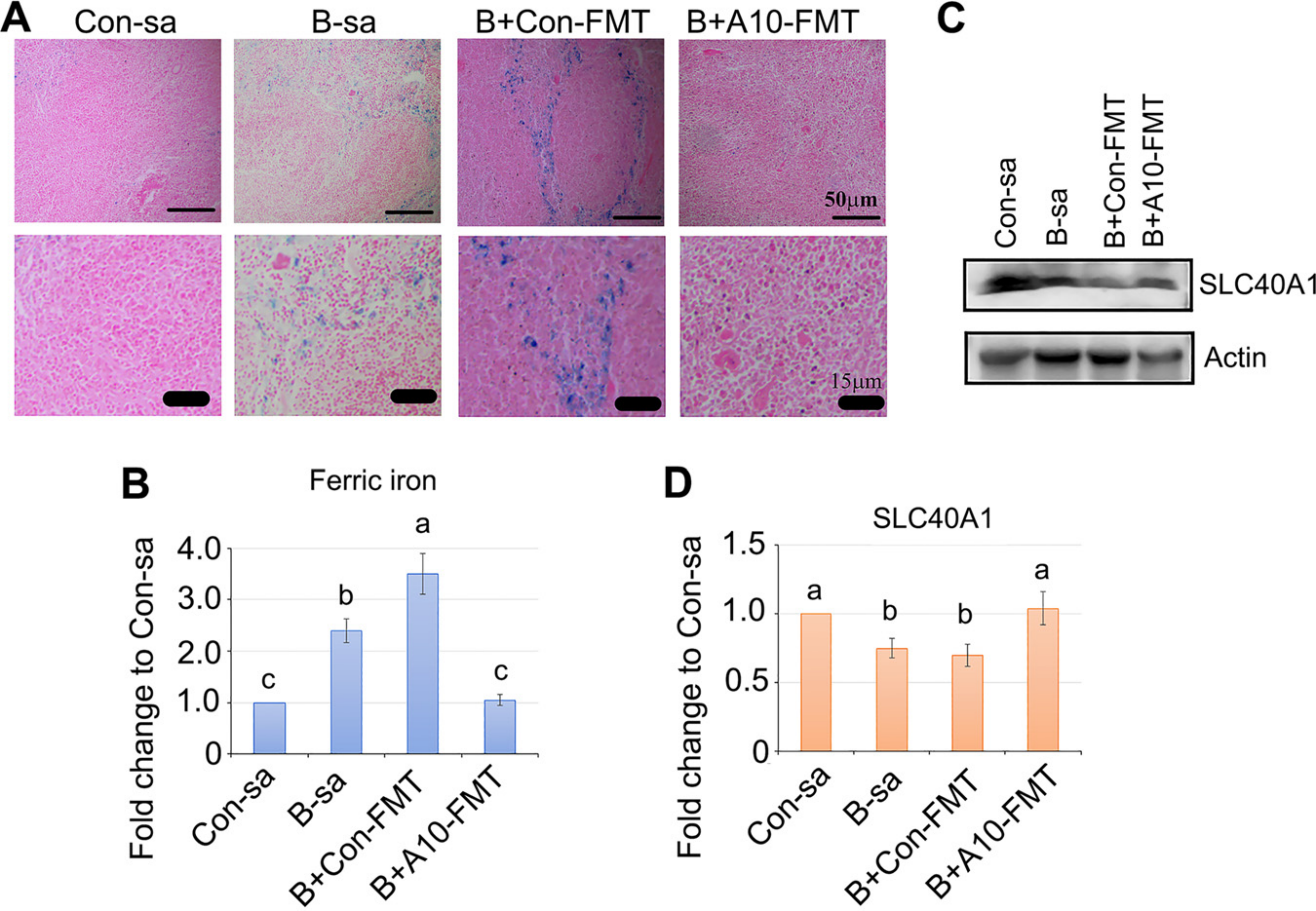
A10-FMT rescued splenic iron homeostasis. (A) Perl’s Prussian blue stain for ferric iron in mouse spleens (*n* = 4 per group). (B) Quantitative data for ferric iron. (C) Protein level of SLC40A1 obtained via WB analysis (*n* = 3 per group). (D) Quantitative data for the WB analysis of SLC40A1. In panels B and D, the *y* axis was the fold change of the Con-sa group. The *x* axis represents the treatments. Note: the a, b, and c notation indicate statistically significant differences between groups with *P* < 0.05.

Moreover, the spleen plays important roles in iron homeostasis via the resorption of effete erythrocytes and the subsequent return of iron to the circulation. Free iron also has the potential to become cytotoxic when electron exchange with oxygen is unrestricted and catalyzes the production of reactive oxygen species. Therefore, the balance of iron in the spleen and circulation is critical for health (49, 50). Busulfan increased the level of iron in the spleen, which was decreased by A10-FMT but not by Con-FMT (Fig. 6A and B). At the same time, the gene expression and protein expression of the iron transporter ferroportin 1 (Slc40a1) were increased by A10-FMT (Fig. 6C–E).

## DISCUSSION

The mammalian gastrointestinal tract is inhabited by millions of microbes that modulate many physiological functions (18, 51–55). An increasing number of diseases, such as obesity (56), cancer (54), inflammatory bowel disease (57), diabetes (19), and male or female infertility (11, 12, 58, 59) have been associated with distinctive changes in the composition and functionality of gut microbiota.

Recently, it has been established that gut microbiota are involved in the development and function of the spleen (13, 14). A gut-spleen interaction (axis) has been identified. However, it remains unknown whether gut microbiota can be used to improve spleen function during cancer therapy or other disease conditions. In our previous articles, using the busulfan- and AOS-dosed gut microbiota models, we found that FMT could benefit recipient gut microbiota to improve blood and testicular metabolites to, in turn, rescue busulfan-impaired spermatogenesis, sperm quality, and male fertility (11). The transplantation of gut microbiota from AOS-dosed animals (A10-FMT) significantly increased sperm motility, sperm concentration, and spermatogenesis. Furthermore, A10-FMT benefited gut microbiota through an increase in the “beneficial” bacteria *Bacteroidales*, *Bifidobacteriales*, and *Sphingomonadales* (11). These microbiota have beneficial effects, including their protection of the intestinal barrier (22, 59), production of antioxidant compounds (60), and possession of reduction enzymes (61). Moreover, A10-FMT improved the blood metabolomes, whereas busulfan disturbed the blood microenvironments by upsetting metabolite levels (11). In the current investigation, we discovered that AOS-improved microbes can ameliorate the anti-cancer drug busulfan-impaired gut microbiota to rescue busulfan-disrupted spleen vascularization and function. The potential mechanisms by which A10-FMT improves spleen vascularization may include that the improved microbiota produce some beneficial molecules to benefit spleen vascularization, that these molecules stimulate systemic beneficial effects to ameliorate spleen function, and that the improved microbiota benefit intestinal cell functions (such as the immune functions) to ameliorate spleen function. We are current investigating the underlying mechanisms.

In conclusion, we demonstrated that following a treatment with AOS (A10-FMT), improved gut microbiota rescued busulfan-disrupted spleen vasculature. A10-FMT improved the expression of genes involved in vascularization, cell proliferation rate, EPC capability, and cell junction molecules to increase vasculature formation in the spleen. The rescue of spleen vasculature restored spleen function by means of improvements in spleen immune cells and in iron metabolism. This may be used as a strategy to minimize the side effects of busulfan, which is an excellent medicine for the treatment of leukemia in children (especially for those who are younger than 3 years old).

## MATERIALS AND METHODS

### Study design.

All animal handling and procedures were performed in accordance with the Institute of Animal Sciences of Chinese Academy of Agricultural Sciences (CAAS) Institutional Animal Care and Use Committee (IACUC). The mice were maintained under a light:dark cycle of 12:12 h at a temperature of 23°C and a humidity of 50% to 70%, and they had free access to food (chow diet) and water (11, 33).

### Experiment I: Mouse small intestine microbiota collection.

3-week-old Institute of Cancer Research (ICR) male mice were dosed with double-distilled water (ddH2O) as a control or with alginate oligosaccharide (AOS), 10 mg/kg body weight (BW), via oral gavage (0.1 mL/mouse/day). The AOS dosing solution was freshly prepared on a daily basis and was delivered every morning for 3 weeks. There were two groups (30 mice/treatment): a control group (ddH2O) and an A10 group (AOS, 10 mg/kg BW). After treatment, the mice were humanely euthanized for the collection of their small intestine luminal content (microbiota) (Fig. 1A).

### Experiment II: Fecal microbiota transplantation (FMT).

The small intestine luminal contents (containing gut microbiota) from each group were pooled and homogenized, diluted 1:1 in 20% sterile glycerol (saline), and frozen. Before inoculation, the fecal samples were diluted in sterile saline to a working concentration of 0.05 g/mL and were filtered through a 70 μm cell strainer. 3-week-old ICR male mice were used in the current investigation. There were four treatment groups (30 mice/treatment): Con-sa (control; dosed with saline), B-sa (busulfan [a single injection of 40 mg/kg BW of busulfan] plus saline), B+Con-FMT (busulfan plus gut microbiota from the control mice [Experiment I]), and B+A10-FMT (busulfan plus gut microbiota from the AOS, 10 mg/kg mice [Experiment I]). The mice received oral FMT inoculations (0.1 mL) once daily for two weeks. The dose of FMT was reported in our previous study and in other reports (11, 19, 62). Moreover, in our preliminary study, we found that 14 days (2 weeks) of FMT can produce a similar effect to that of 35 days (5 weeks) of FMT and a better effect than that of 7 days (1 week) of FMT. The mice were maintained with regular care for another 3 weeks (to 8 weeks of age) and were then humanely euthanized for the collection of samples for different analyses. The animal experiments were done more than three times (Fig. 1A). The animals in this study were the same animals as those published in our earlier study (11). The spleen tissues were used in the current study, and others, such as the intestinal content (gut microbes), blood (metabolites), and testes (spermatogenesis), were determined and reported in our earlier article (11). In order to get a sufficient quantity of samples for all of the analyses (especially for the spermatogenesis), 30 male animals were used in each treatment group. However, for the Western blotting, RNA-seq, immunofluorescence staining, and other analyses, 3 to 6 samples were used for each group. There were 15 to 20 samples used in the gut microbiota and blood metabolites analyses.

### Ultrastructural analysis of spleen tissues via transmission electron microscopy (TEM).

The procedure for the TEM analysis has been reported in our earlier article (63). Briefly, the freshly collected intestinal tissue was fixed in 2% glutaraldehyde in sodium phosphate buffer (pH 7.2) for 2 h. The samples were then washed 5 times in phosphate buffer (PBS) and fixed in 1% OsO_4_ for 1 h in the dark. This was followed by several washes in PBS. Subsequently, the samples were dehydrated in ethanol, using a series of increasing concentrations, and infiltrated in Spur’s embedding medium in propylene epoxide with an increased concentration. Afterwards, the specimens were polymerized for 12 h at 37°C, 12 h at 45°C, and 48 h at 60°C in embedding medium. 50 nm sections were cut using a Leica Ultracut E that was equipped with a diamond knife (Diatome, Hatfield, PA, USA). The sections were stained with uranyl acetate and were viewed on a microscope (JEM-2010F TEM; JEOL Ltd., Japan).

### RNA isolation and RNA-seq analyses.

Briefly, the total RNA was isolated using TRIzol Reagent (Invitrogen) and was purified using a Pure-Link1 RNA Mini Kit (Cat: 12183018A; Life Technologies), following the manufacturers’ protocol. The sequencing and data analyses are reported in our earlier article (33). The total RNA samples were first treated with DNase I to degrade any possible DNA contamination. Then, the mRNA was enriched using oligo(dT) magnetic beads. Mixed with the fragmentation buffer, the mRNA was broken into short fragments (of about 200 bp), after which the first strand of cDNA was synthesized using a random hexamer-primer. Buffer, dNTPs, RNase H, and DNA polymerase I were added to synthesize the second strand. The double-strand cDNA was purified with magnetic beads. Subsequently, the 3′-end single nucleotide adenine addition was performed. Finally, sequencing adaptors were ligated to the fragments. The fragments were enriched via polymerase chain reaction (PCR) amplification. During the QC step, an Agilent 2100 Bioanaylzer and an ABI StepOnePlus real-time PCR system were used to qualify and quantify the sample library. The library products were prepared for sequencing in an Illumina HiSeq 2500. The reads were mapped to reference genes using SOAPaligner (v. 2.20) with a maximum of two nucleotide mismatches allowed at the parameters of “-m 0 -x 1000 -s 40 -l 35 -v 3 -r 2”. The read number of each gene was transformed into reads per kilobases per million reads (RPKM), and then differentially expressed genes were identified using the DEGseq package and the MARS (an MA-plot-based method with a random sampling model) method. The threshold was set as a false detection rate (FDR) of ≤0.001 and an absolute value of the log_2_ ratio of ≥1 to judge the significance of the differences in gene expression. Then, the data were analyzed via GO enrichment and KEGG enrichment, and multiple enrichment was determined online (Metascape: http://metascape.org/gp/index.html#/main/step1).

### Sequencing of microbiota from small intestine digesta samples and data analysis.

The sequencing and analysis of the gut microbiota are reported in our earlier study (33).

**(i) DNA extraction.** The total genomic DNA of the small intestine digesta was isolated using an E.Z.N.A.R. Stool DNA Kit (Omega Bio-tek Inc., USA), following the manufacturer’s instructions. DNA quantity and quality were analyzed using a NanoDrop 2000 (Thermo Scientific, USA) and 1% agarose gel. 10 samples/groups were determined.

**(ii) Library preparation and sequencing.** The V3-V4 region of the 16S rRNA gene was amplified using the primers MPRK341F (5′- ACTCCTACGGGAGGCAGCAG-3′) and MPRK806R: (5′-GGACTACHVGGGTWTCTAAT-3′) with Barcode. The PCRs (30 μL in total) included 15 μL PhusionR High-Fidelity PCR Master Mix (New England Biolabs), 0.2 mM primers, and 10 ng DNA. The thermal cycle was carried out with an initial denaturation at 98°C, and this was followed by 30 cycles of 98°C for 10 s, 50°C for 30 s, 72°C for 30 s, and a final extension at 72°C for 5 min. The PCR products were purified using a GeneJET Gel Extraction Kit (Thermo Scientific, USA). The sequencing libraries were constructed with a NEB NextR Ultra DNA Library Prep Kit for Illumina (NEB, United States), following the manufacturer’s instructions. Index codes were added. Then, the library was sequenced on an Illumina HiSeq 2500 platform, and 300 bp paired-end reads were generated at the Novo gene. The paired-end reads were merged using FLASH (V1.2.71). The quality of the tags was controlled in QIIME (V1.7.02). Meanwhile, all chimeras were removed. The “Core Set” of the Greengenes database was used for classification, and sequences with >97% similarity were assigned to the same operational taxonomic units (OTUs).

**(iii) Analysis of sequencing data.** The operational taxonomic unit abundance information was normalized using a standard sequence number that corresponded to the sample with the least sequences. The alpha diversity index was calculated with QIIME (Version 1.7.0). The Unifrac distance was obtained using QIIME (Version 1.7.0), and a principal coordinates analysis (PCoA) was performed using R (Version 2.15.3). The linear discriminant analysis effect sizes (LEfSe) were calculated to determine the differences in abundance. The threshold linear discriminant analysis (LDA) score was 4.0. The GraphPad Prism7 software package was used to produce the graphs.

### Plasma metabolite measurements by liquid chromatography - mass spectrum (LC-MS/MS).

Plasma metabolites were determined as reported in our recent article (33). The plasma samples were collected and immediately stored at −80°C. Prior to the LC-MS/MS analysis, the samples were thawed on ice and processed to remove proteins. Then, the samples were detected via ACQUITY ultra-performance liquid chromatography (UPLC) and AB Sciex Triple TOF 5600 (LC/MS) as reported previously. 10 samples/groups were analyzed for the plasma or testis samples.

The high performance liquid chromatography (HPLC) conditions employed an ACQUITY UPLC BEH C18 column (100 mm × 2.1 mm, 1.7 μm), using solvent A (aqueous solution with 0.1% [vol/vol] formic acid) and solvent B (acetonitrile with 0.1% [vol/vol] formic acid) with a gradient program. Further details are shown in Table 1. The flow rate was 0.4 mL/min, and the injection volume was 5 μL. The mass spectrometry program with ESI is shown in Table 2.

**TABLE 1:** HPLC elution gradient

Time	A%	B%
0	95	5
2	80	20
4	75	25
9	40	60
17	0	100
19	0	100
19.1	95	5
20.1	95	5

**TABLE 2:** Mass spectrometry conditions

Parameters	Positive ion	Negative ion
Nebulizer gas (GS1, PSI)	40	40
Auxiliary gas (GS2, PSI)	40	40
Curtain gas (CUR, PSI)	35	35
Ion source temperature (°C)	550	550
Ion spray voltage (V)	5,500	4,500
Declustering potential (DP, V)	100	−100
Mass scan range (TOF MS scan)	70 to 1,000	70 to 1,000
Collision energy (TOF MS scan, eV)	10	−10
Mass scan range (Product Ion scan)	50 to 1,000	50 to 1,000
Collision energy (Product Ion scan, eV)	30	30
Interface heater temperature (°C)	550	600

Progenesis QI v2.3 (Nonlinear Dynamics, Newcastle, United Kingdom) was implemented to normalize the peaks. Then, the Human Metabolome Database (HMDB), LIPID MAPS (v2.3), and METLIN software were used to qualify the data. Moreover, the data were processed with SIMCA software (version 14.0, Umetrics, Umeå, Sweden) after the pathway enrichment analysis using the Kyoto Encyclopedia of Genes and Genomes (KEGG) database (https://www.genome.jp/kegg/pathway.html).

### Histopathological analysis.

The spleen tissues were fixed in 10% neutral buffered formalin, embedded in paraffin, cut into 5 μm sections, and subsequently stained with hematoxylin and eosin (H&E) for the histopathological analysis (33).

### Western blotting.

A Western blotting analysis of the proteins was carried out as previously reported (33). Briefly, small intestine tissue samples were lysed in RIPA buffer that contained the protease inhibitor cocktail from Sangong Biotech, Ltd. (Shanghai, China). The protein concentration was determined using a BCA kit (Beyotime Institute of Biotechnology, Shanghai, China). Goat anti-actin was used as a loading control. The remaining primary antibodies (Abs) were purchased from Abcam or Beijing from Biosynthesis Biotechnology Co., Ltd., (Beijing, China) (Table S1). Secondary donkey anti-goat Abs (Cat no.: A0181) were purchased from the Beyotime Institute of Biotechnology, and goat anti-rabbit (Cat no.: A24531) Abs were bought from Novex by Life Technologies (USA). 50 μg of total protein per sample were loaded onto 10% sodium dodecyl sulfate (SDS) polyacrylamide electrophoresis gels. The gels were transferred to a polyvinylidene fluoride (PVDF) membrane at 300 mA for 2.5 h at 4°C. The membranes were then blocked with 5% bovine serum albumin (BSA) for 1 h at room temperature (RT), and this was followed by three washes with 0.1% Tween 20 in TBS (TBST). The membranes were incubated with primary Abs that were diluted 1:500 in TBST with 1% BSA overnight at 4°C. After three washes with TBST, the blots were incubated with the HRP-labeled secondary goat anti-rabbit or donkey anti-goat Abs, respectively, for 1 h at RT. After three washes, the blots were imaged. The bands were quantified using ImageJ software. The intensity of the specific protein band was normalized to actin first, and then the data were normalized to the control. The information for the primary antibodies is listed in Table S1.

10.1128/msphere.00581-22.2 TABLE S1 Primary antibody information. Download Table S1, DOCX file, 0.02 MB. Copyright © 2022 Fang et al. 2022 Fang et al. https://creativecommons.org/licenses/by/4.0/ This content is distributed under the terms of the Creative Commons Attribution 4.0 International license.

### Detection of the protein levels and the location in the spleen via immunofluorescence staining.

The methodology for the immunofluorescence staining of the spleen samples is reported in our recent publications (11, 33). Sections of testicular tissue (5 μm) were prepared and subjected to antigen retrieval and immunostaining as previously described. Briefly, sections were first blocked with normal goat serum in PBS, and this was followed by incubation with primary Abs (Table S1) (1:100 in PBS-0.5% Triton X-100; Bioss Co., Ltd., Beijing, People’s Republic of China) overnight at 4°C. After a brief wash, the sections were incubated with an Alexa 546-labeled goat anti-rabbit secondary Ab (1:100 in PBS; Molecular Probes, Eugene, OR, USA) for 30 min at RT and were then counterstained with 4′,6-diamidino-2-phenylindole (DAPI). The stained sections were examined using a Leica Laser Scanning Confocal Microscope (LEICA TCS SP5 II, Germany). 10 animal samples from each treatment group were analyzed. The positively stained cells were counted. A minimum of 1,000 cells were counted for each sample of each experiment. The data were then normalized to the control. The information for the primary antibodies is listed in Table S1.

### Measurement of the iron content in the spleen.

The amounts of ferric iron in the spleens were determined using Perl’s Prussian blue stain as described by Kohyama et al. (40). The spleen tissues were fixed with 4% paraformaldehyde and were then embedded in paraffin. 5 μm sections were cut and stained with Perl’s Prussian blue and pararosaniline (Sigma-Aldrich; St. Louis, MO, USA).

### Statistical analysis.

Data were analyzed using SPSS statistical software (IBM Co., NY, USA) with one-way analysis of variance (ANOVA) followed by LSD multiple-comparison tests. All groups were compared with each other for every parameter. The data were shown as the mean ± SEM. Statistical significance was based on *P* < 0.05.

### Data availability.

The RNA-seq raw data are deposited in NCBI’s Gene Expression Omnibus under accession number GSE160965.
