# Supplementary figures and images for: Gut-Spleen Axis: Microbiota via Vascular and Immune Pathways Improve Busulfan-Induced Spleen Disruption
Source: mSphere. 2022 Dec 13;8(1):e00581-22. doi: 10.1128/msphere.00581-22 (PMC9942571; doi:10.1128/msphere.00581-22)

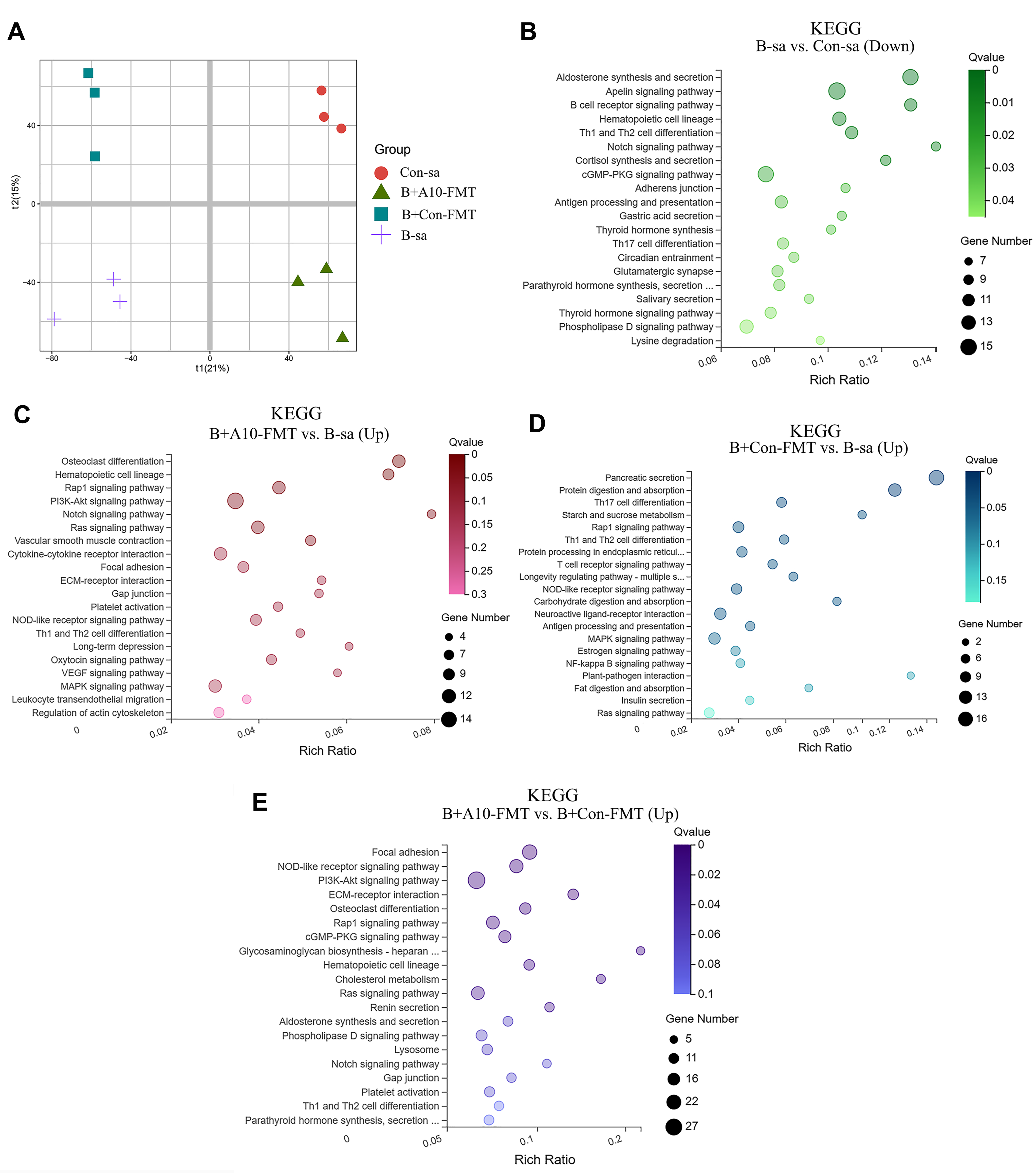

Supplement: FIG S1 [file msphere.00581-22-s0001.tif]
