# Supplementary material for: Gut-Spleen Axis: Microbiota via Vascular and Immune Pathways Improve Busulfan-Induced Spleen Disruption
Source: mSphere. 2022 Dec 13;8(1):e00581-22. doi: 10.1128/msphere.00581-22 (PMC9942571; doi:10.1128/msphere.00581-22)
Supplement: TABLE S1 [file msphere.00581-22-s0002.docx]

Primary antibody information

| **Gene symbol** | **Name** | **Cat. #** | **Predicted size** | **Source (Animal)** | **Company** |
| --- | --- | --- | --- | --- | --- |
| Bcl-2 | Bcl-2 | bs-4563R | 26kd | Rabbit (polyclonal) | Beijing Biosynthesis Biotechnology CO. |
| Ki67 | Antigen identified by monoclonal antibody Ki 67 | bs-2130R | 358kd | Rabbit (polyclonal) | Beijing Biosynthesis Biotechnology CO. |
| P53 | Transformation related protein 53 | bs-8687R | 53kd | Rabbit (polyclonal) | Beijing Biosynthesis Biotechnology CO. |
| CD31 | CD31 | bs-0195R | 78kDa | Rabbit | Beijing Biosynthesis Biotechnology CO. |
| VEGF Receptor 2 | VEGF Receptor 2 | ab194806 | 152 kDa | Rabbit | Abcam |
| CD163 | CD163 | bs-2527R | 130kDa | Rabbit | Beijing Biosynthesis Biotechnology CO. |
| MAdCAM1 | MAdCAM1 | ab254539 |  | Rat | Abcam |
| VCAM1 | VCAM1 | ab134047 | 81 kDa | Rabbit | Abcam |
| Cx43 | Connexin 43 | bs-0651R | 42kDa | Rabbit (polyclonal) | Beijing Biosynthesis Biotechnology CO. |
| CCR7 | CCR7 | bs-1305R | 42kDa | Rabbit | Beijing Biosynthesis Biotechnology CO. |
| CCL21 | CCL21 | bs-1666R | 15kDa | Rabbit | Beijing Biosynthesis Biotechnology CO. |
| actin | actin | Ab3280 | 42kDa | Rabbit (polyclonal) | Abcam |
| SLC40A1 | SLC40A1 | ab78066 |  | Rabbit | Abcam |
| CD27 | CD27 | bsm-54318R | 55kDa | Rabbit | Beijing Biosynthesis Biotechnology CO. |
| p75 NGF Receptor | p75 NGF Receptor | bs-7122R | 42kDa | Rabbit | Beijing Biosynthesis Biotechnology CO. |
| MOMA | MOMA | bs-17487R | 65kDa | Rabbit | Beijing Biosynthesis Biotechnology CO. |
| CD59/Ly6c | CD59/Ly6c | bs-12327R | 9kDa | Rabbit | Beijing Biosynthesis Biotechnology CO. |
